# The Role of Gerotranscendence Theory and Physical, Psychological, and Social Determinants in Predicting Life Satisfaction: A Structural Equation Modeling Analysis

**DOI:** 10.3390/healthcare13212787

**Published:** 2025-11-03

**Authors:** Yadollah Abolfathi Momtaz, Farahnaz Mohammadi-Shahboulaghi, Masoumeh Alijanpour, Atefeh Omrani, Raziye Sadat Hosseini

**Affiliations:** 1 Iranian Research Center on Aging, University of Social Welfare and Rehabilitation Sciences, Tehran 19857-13871, Iran; yabolfathi@gmail.com (Y.A.M.); f.mohammadi@uswr.ac.ir (F.M.-S.); 2 Research Institute on Ageing (MyAgeing), Universiti Putra Malaysia, Serdang 43400, Malaysia; 3 Infertility and Reproductive Health Research Center, Health Research Institute, Babol University of Medical Sciences, Babol 47176-41367, Iran; masoomalijanpoor@yahoo.com; 4 Department of Health and Social Care, Faculty of Education and Society, University of Sunderland in London, London E14 9GE, UK; atefehomrani@outlook.com; 5 Geriatric Mental Health Research Center, School of Behavioral Sciences and Mental Health, Tehran Institute of Psychiatry, Iran University of Medical Sciences, Tehran 14496-14535, Iran

**Keywords:** gerotranscendence theory, life satisfaction, social determinant, psychological determinant, physical determinant

## Abstract

Background: (1) Objective: This study aimed to develop and evaluate a structural model of life satisfaction in older adults, integrating the World Health Organization’s biopsychosocial determinants and the theory of gerotranscendence to provide a more holistic understanding of the aging experience. (2) Methods: A cross-sectional study was conducted with 600 older adults (≥60 years) residing in Tehran, selected through proportional random sampling. Data was collected via validated instruments assessing demographic factors, physical and mental health, social support, environmental condition quality, and gerotranscendence. Structural Equation Modeling (SEM) using AMOS 24 and hierarchical regression were employed for data analysis. (3) Results: The findings revealed that physical health, mental health, social support, environmental conditions, and gerotranscendence were all significantly associated with life satisfaction (*p* < 0.01). Hierarchical regression analysis showed that gerotranscendence remained an independent and significant predictor of life satisfaction, even after accounting for other variables. The final model explained approximately 39% of the variance in life satisfaction and demonstrated good fit indices (RMSEA = 0.051, CMIN/DF = 2.545, PCLOSE = 0.272, GFI = 0.815). (4) Conclusion: The proposed model offers a comprehensive framework for understanding life satisfaction in older adults, highlighting the unique contribution of gerotranscendence. These findings highlight the importance of integrated interventions that enhance physical and mental health, promote social and environmental well-being, and foster spiritual growth. Future research should consider longitudinal and mixed-method designs to further explore causal relationships and cultural contexts.

## 1. Introduction

Census data in Iran indicate a steadily growing trend in the elderly population. Over approximately 42 years, the proportion of older adults in Iran has doubled, from 5% in 1976 to 10% in 2019, and is projected to double again, reaching 20% by 2041 [1]. In contrast, in many developed countries, this demographic shift has occurred over more than a century. Iran is therefore among the countries experiencing one of the steepest and fastest increases in its aging population worldwide [1]. While population ageing is considered one of the major achievements of human, health, and social development [2], this demographic shift also presents a range of complex, multifaceted challenges that impact multiple sectors of society. In many communities, aging continues to be perceived through stereotypical and largely negative perspectives, often depicting older adults as passive, vulnerable, socially isolated, and no longer contributing meaningfully to society [3]. This cumulative decline simultaneously exposes older adults to health-related vulnerabilities and an increased risk of medical complications. Moreover, research consistently shows a marked decline in objective quality of life with increasing age, as indicators of functional ability and financial well-being tend to deteriorate in older populations [4,5,6]. Despite the well-documented positive association between life satisfaction and health, socioeconomic status, and psychological factors [7,8], multiple studies suggest that subjective well-being and overall life satisfaction do not necessarily decline with age. In fact, despite the physical and social losses commonly linked to ageing, many older adults report stable or even enhanced levels of well-being. This apparent paradox is largely attributed to the development of emotional regulation competencies and the active use of adaptive emotional strategies. These include fostering positive emotional experiences and optimally utilizing social support networks, which in turn enhance psychological and social resilience [9]. Empirical research supports the concept of the “ageing paradox”: despite experiencing cognitive, social, and physical decline, as well as the challenges of age-related crises, life satisfaction, the cognitive dimension of subjective well-being, representing the balance between aspirations and present circumstances, often remains stable, with no statistically significant decline observed among older adults [7,10].

According to Veenhoven and colleagues, life satisfaction is a multidimensional construct that includes psychological well-being, physical health, occupational functioning, social relationships, economic status, and daily functioning [11]. It is also shaped by adaptive processes and an individual’s perception of their position within their cultural context, as well as the alignment between their values, goals, expectations, and personal interests. Other studies have associated life satisfaction with factors such as emotional states, mood, feelings, spiritual beliefs, and a sense of purpose in life [12]. Overall, life satisfaction is best understood as a multidimensional construct, especially in later life, encompassing physical and mental health, socioeconomic status, social and family relationships, and the quality of the living environment [13].

Although Tornstam’s theory of gerotranscendence provides a valuable framework for understanding positive psychological development in late life, its empirical validation has predominantly emerged from Western cultural contexts [14]. This presents a significant gap, as the pathway to and expression of gerotranscendence are likely deeply mediated by cultural norms, spiritual beliefs, and social structures. The present study seeks to address this gap by examining gerotranscendence within the unique socio-cultural fabric of Iran, a representative Middle Eastern society [15]. Iranian culture is characterized by a rich tapestry of Islamic and Sufi spiritual traditions, strong intergenerational family bonds, and collectivistic values that emphasize familial duty and respect for elders. These elements may profoundly shape how older adults reconfigure their relationships with the self, others, and the cosmos—the core dimensions of gerotranscendence [16]. Therefore, this research is not merely an application of the theory in a new setting, but an investigation into how its universal principles is articulated through the specific lenses of spirituality, family, and community in the Iranian context, thereby contributing to a more cross-culturally nuanced understanding of successful aging.

**Figure 1:**
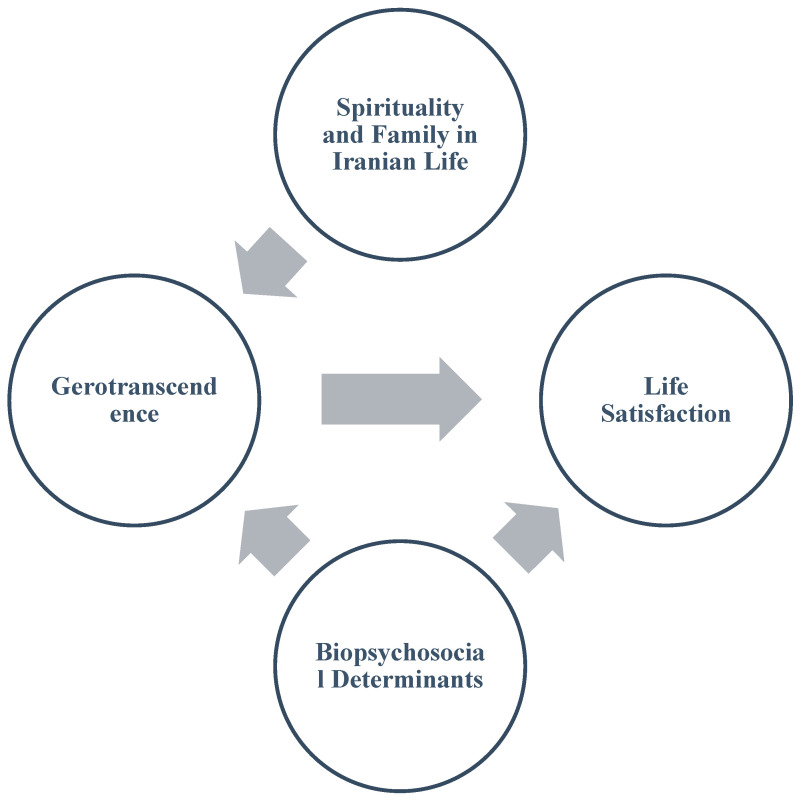
Conceptual Framework Diagram.

A review of life satisfaction models shows that no single framework yet captures all the factors influencing it [17]. Existing theories and models often focus on specific dimensions, lacking a holistic perspective on the phenomenon. As such, these models are open to critique, particularly when applied to understanding life satisfaction in older adults. Therefore, developing a conceptual model that provides a more integrated and comprehensive approach to life satisfaction is essential to improve the quality of life for the elderly. In this study, the researchers have developed a model integrating life satisfaction factors identified by the World Health Organization (WHO) (Figure 1). They assessed its goodness of fit and refined it using gerotranscendence theory, with the aim of creating a more comprehensive, multidimensional framework for understanding life satisfaction in older adults.

## 2. Materials and Methods

### 2.1. Study Design

This was a cross-sectional study utilizing a structural equation modeling (SEM) approach. Structural Equation Modeling (SEM) was selected as the primary analytical method for several key reasons, all of which are particularly pertinent to gerontological research. First, our study involves several key concepts (e.g., mental health, social support, gerotranscendence) that are latent variables, meaning they cannot be directly observed but are inferred from a set of measured indicators. SEM is powerful because it incorporates this measurement model, accounting for random error and providing a more valid estimate of the underlying constructs. Second, gerontological phenomena are inherently complex, often involving interconnected pathways. SEM allows for the simultaneous testing of all hypothesized direct and indirect relationships within our integrated biopsychosocial–spiritual model, offering a comprehensive view that simpler statistical methods (like multiple regression) cannot provide [18]. The study sample included older adults aged ≥60 years residing in Tehran, who were selected through proportional random sampling from six urban districts (1, 4, 5, 9, 15, and 19), based on the geographical distribution of the target population. Rather than using a relative power analysis, the sample size was estimated according to the ratio of the number of participants to the number of free parameters in the model. It is recommended that this ratio be at least 5:1, with an average of 10:1 and an upper limit of 20:1 [19,20]. Based on the 112 items included in the questionnaire, the minimum required sample size was estimated at 560 (5 × 112). Accounting for a potential 20% attrition rate, a final sample of 600 eligible older adults was recruited. Inclusion criteria were age ≥60 years, residence in Tehran, verbal communication ability, no use of cognition-impairing medications, and absence of terminal illnesses affecting response ability. Exclusion criteria included withdrawal, incomplete questionnaires, and the onset of acute physical or psychological conditions during the study.

Data collection began with telephone screening based on an available list of phone numbers. Using telephone directories, phone numbers were randomly selected. This screening was conducted by trained interviewers to assess inclusion criteria and obtain verbal consent. Two trained interviewers, who had previously collaborated in multiple studies, were uniformly trained by the researcher on data collection and questioning techniques. To ensure the quality of data collection, standardized interviewer training was provided, achieving an inter-rater agreement of ≥0.85.

Initially, trained interviewers contacted potential participants via telephone to explain the study objectives. If the older adult met the inclusion and exclusion criteria, verbal consent was obtained over the phone. If not, another number was contacted until the target sample size for each district was reached. In District 19, 8 older adults, and in District 9, 21 older adults, were unresponsive or declined participation. Replacement phone numbers were randomly provided by the health centers.

After obtaining verbal consent, the researcher or trained interviewers visited participants’ homes at scheduled times to administer the questionnaires in person. If multiple eligible individuals were present at one address, one was randomly selected to reduce intra-group correlation. The 112-item questionnaire took approximately 40–50 min to complete. Questionnaires were completed over multiple sessions if necessary. As the questionnaires were self-reported, the responses provided by the participants to the interviewers were recorded. Participants were assured of confidentiality and their right to withdraw at any time. They were also informed that they could pause the questionnaire if fatigued and resume at a later time. This process continued until the target statistical sample size was achieved. With this approach, *all 600 participants who were recruited completed the entire set of questionnaires, resulting in a complete dataset with no missing values for the analyzed items.* The final analysis was therefore conducted on complete data from all 600 participants.

### 2.2. Instruments

A researcher-developed demographic questionnaire was used to collect baseline data. It included 12 multiple-choice items covering year of birth, gender, ethnicity, marital status, number of children, literacy, education, employment status, income source, home ownership, use of mobility aids, and presence of a caregiver. The questionnaire’s validity and reliability were established following standard design principles. To assess life satisfaction, the Life Satisfaction Index-Z (LSI-Z) developed by Wood, Wylie, and Sheafor (1969) was used [21]. This tool contains 13 items, each scored on a 0–2 scale (total score range: 0–26), with higher scores indicating greater life satisfaction. The construct validity of the instrument was confirmed using the known-groups method, and its reliability was established with a Cronbach’s alpha of 0.79. The Persian version was validated by Taghribi et al. (2009) [22].

Self-Rated Health (SRH) was also used to assess participants’ subjective perception of their health status. This was measured through a single item in three formats: non-comparative, comparative to the past, and comparative to peers. This tool demonstrated concurrent validity with the Charlson Comorbidity Index (CCI) (r = −0.35) and the WHO-5 Well-Being Index (r = 0.50). Its reliability was confirmed through test–retest correlation (r = 0.76), and its Persian version was validated by Hosseini and colleagues [23].The Charlson Comorbidity Index (CCI) was used to assess disease burden and mortality risk, considering 19 medical conditions across four levels (scoring from 1 to 6) [24]. The total score ranges from 0 to 37, with higher scores indicating more severe comorbidity. The validity of this tool was confirmed through concurrent validity with the Activities of Daily Living (ADL) scale (r = −0.22), and its reliability was established via test–retest reliability (r = 0.64) [25]. The Activities of Daily Living (ADL) Questionnaire was used to assess physical functioning and measure independence in personal and household activities [26]. The scoring system ranges from 0 to 5, where 0–1 indicates dependence, 2–3 partial independence, and 4–5 full independence. This instrument has been validated in Iran, with content validity (CVR = 0.82), sensitivity (0.75), specificity (0.96), and a Cronbach’s alpha reliability coefficient of 0.80 [27].

To assess psychological well-being, the World Health Organization Five Well-Being Index (WHO-5) was used [28]. This instrument includes 5 items rated on a 6-point Likert scale (total score range: 0–25). A score of less than 13 was considered indicative of poor well-being. The WHO-5 has demonstrated good convergent validity with the Oxford Happiness Questionnaire (r = 0.57) and divergent validity with the Beck Depression Inventory (r = −0.61). It also showed high reliability in the Iranian population, with a Cronbach’s alpha of 0.89 [29].

To assess cognitive status, the Abbreviated Mental Test (AMT) developed by Hodkinson (1972) was used [30]. This tool consists of 10 questions and is designed for screening cognitive impairments. It has shown good sensitivity (92.15%), specificity (81.5%), and acceptable reliability, indicated by a Cronbach’s alpha of 0.76 [31].

The Medical Outcomes Study Social Support Survey (MOS-SSS), developed by Sherbourne and Stewart (1991), was used to assess the social support status of older adults [32,33]. This instrument contains 19 items across four subscales: emotional support, informational support, tangible support, and positive social interaction. The total score ranges from 19 to 95, with higher scores indicating greater perceived social support. The Persian version was psychometrically evaluated by Alavizadeh et al. (2024), confirming concurrent validity and high internal consistency (Cronbach’s alpha = 0.93) [34].

In addition, the Lubben Social Network Scale (LSN-6) was employed to measure perceived social support from family and friends [35]. This 6-item scale yields a total score ranging from 0 to 30, where higher scores indicate stronger social support. The LSN-6 demonstrated acceptable concurrent validity with the MOS-SSS (r = 0.33), as well as good reliability, with Cronbach’s alpha = 0.72 and test–retest reliability = 0.90 [36].

To assess the quality of the physical environment, the World Health Organization Quality of Life—BREF (WHOQOL-BREF) tool was used, specifically the domain on environmental conditions [37]. This section includes 8 items with a total score ranging from 4 to 20, where higher scores indicate a more favorable environment. The instrument demonstrated good reliability, with Cronbach’s alpha values of 0.84 in older adults and 0.72 in patient populations. The validity and reliability of the Persian version were confirmed by Nejati and colleagues in 2006 [38].

Finally, the Gerotranscendence Scale by Cozort (2008) was used to measure levels of gerotranscendence [39]. This scale consists of 25 items rated on a 4-point Likert scale and covers three dimensions: cosmic transcendence, self, and social relations. Higher scores indicate greater levels of gerotranscendence. The Persian version was psychometrically evaluated by Hosseini and colleagues in 2019, demonstrating acceptable predictive validity (r = 0.46) and reliability (Cronbach’s alpha = 0.84; test–retest reliability = 0.70) [36].

### 2.3. Statistical Analysis

Data analysis was conducted in multiple stages using SPSS Statistics version 23 and AMOS version 24 employing the Maximum Likelihood (ML) estimation method. Descriptive Statistics conducted for frequencies, percentages, means, and standard deviations were calculated for all demographic and clinical variables. Bivariate Analyses carried out by Pearson correlation coefficients were computed to examine relationships between continuous variables (e.g., life satisfaction with physical health, mental health, social support, environmental conditions, and gerotranscendence). Indeed, independent *t*-tests and ANOVA were used to compare group differences where appropriate. For multivariate analyses, hierarchical regression analysis was performed to examine the unique contribution of gerotranscendence in predicting life satisfaction after controlling for demographic and biopsychosocial variables. R^2^ change values were calculated to determine the variance explained at each step. Structural Equation Modeling (SEM) using Maximum Likelihood estimation was employed to test the comprehensive theoretical model. Finally, model fit was assessed using multiple indices: CMIN/DF, RMSEA, GFI, and PCLOSE. In terms of statistical significance, all tests were two-tailed with the alpha set at 0.05.

## 3. Results

The results showed that the mean age of the older adults was 76.82 ± 7.45 years. The majority were women (63.7%), of Fars ethnicity (57.1%), and married (63.1%). Over 47.0% of the elderly participants reported using at least one assistive mobility device, and approximately 59.0% indicated that they had a caregiver.

Statistical analysis showed that life satisfaction was associated with several variables used in the study. Pearson correlation coefficients indicated a significant relationship between life satisfaction and physical health indicators (CCI, SRH, ADL) (*p* < 0.001). Specifically, higher scores in Activities of Daily Living (ADL) and Self-Rated Health (SRH) were associated with greater life satisfaction among the older adults. Conversely, the negative correlation with the Charlson Comorbidity Index (CCI) indicated an inverse relationship, meaning that higher levels of comorbidity were associated with lower life satisfaction.

Life satisfaction was also significantly associated with mental health (WHO-5 Well-Being Index and AMT) (*p* < 0.01), social health (MOS-SSS and LSN-6) (*p* < 0.01), environmental conditions (WHOQOL-BREF) (*p* < 0.01), and gerotranscendence (*p* < 0.01). In other words, the higher the participants scored on the questionnaires related to mental well-being, social support, environmental conditions, and gerotranscendence, the higher their reported life satisfaction (Table 1).

Table 2 presents the results of the hierarchical regression analysis. As shown in the table, the first block, comprising demographic variables including age, gender, ethnicity, marital status, education level, living arrangement, employment status, and income, was significantly associated with life satisfaction (R^2^ change = 0.040, *p* < 0.01).

In the second block, variables related to physical health, mental health, social health, and environmental condition also showed a significant association with life satisfaction, independent of demographic variables. On the other hand, biopsychosocial determinants demonstrated that mental health (WHO-5) and environmental conditions (WHOQOL) were the most significant predictors, with standardized coefficients of β = 0.360 and β = 0.170, respectively (R^2^ change = 0.330, *p* < 0.01).

The addition of gerotranscendence in Block 3 provided a statistically significant increment in explained variance (R^2^ change = 0.020, *p* < 0.01), indicating its unique contribution beyond the other variables. This suggests that while demographic and biopsychosocial factors form the foundation of life satisfaction, gerotranscendence adds a distinct dimension to the model. Finally, the key variable of interest, gerotranscendence, was entered in the third block. In total, the combined R^2^ change from all three blocks (0.040 + 0.330 + 0.020) indicates that approximately 39% of the variance in life satisfaction could be explained by the variables examined in this study, representing a relatively substantial proportion.

**Figure 2:**
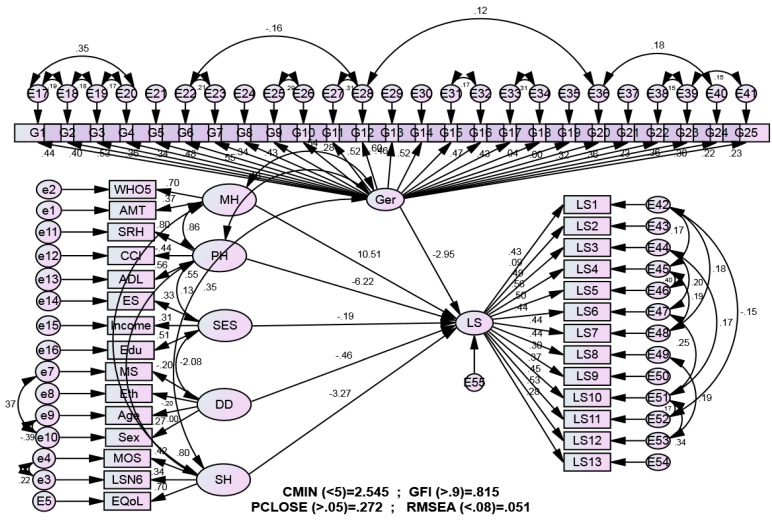
Structural Equation Model of Life Satisfaction in Older Adults following hierarchical regression analysis. Note. Standardized path coefficients are shown. All paths are significant at *p* < 0.05. SRH = Self-Rated Health; CCI = Charlson Comorbidity Index; ADL = Activities of Daily Living; WHO-5 = World Health Organization Five Well-Being Index.

Table 3 presents the results of selected indices used to report the goodness-of-fit of the proposed model, which integrates physical health, mental health, social health, environmental condition, and gerotranscendence as predictors of life satisfaction among older adults. As indicated in Table 3, according to the standard benchmarks discussed in the literature (as previously mentioned), the values obtained support the proposed model, suggesting that it has an acceptable level of goodness-of-fit. Pearson correlation coefficients showed that physical health, mental health, social health, environmental condition, and gerotranscendence were significantly associated with life satisfaction (r = 0.170 to 0.550, *p* < 0.01). Hierarchical regression analysis demonstrated that, beyond the influence of the other variables, gerotranscendence was independently associated with life satisfaction (R^2^ change = 0.020, *p* < 0.01). The structural model testing the integrated relationships between all variables is presented in Figure 2. The model demonstrates the standardized path coefficients between gerotranscendence, physical health, mental health, social support, environmental conditions, and life satisfaction. All paths shown were statistically significant (*p* < 0.05) and support the hypothesized relationships in our conceptual framework. Indeed, the model fit indices indicated a good fit between the proposed model and the data, with RMSEA = 0.051, CMIN/DF = 2.545, PCLOSE = 0.272, and GFI = 0.815.

## 4. Discussion

The findings of this study demonstrate a significant relationship between gerotranscendence development and life satisfaction among Iranian older adults. Our results indicate that the integrated model of WHO health determinants and gerotranscendence theory effectively predicted life satisfaction, accounting for approximately 39% of the variance in life satisfaction score. The assessment of life satisfaction serves as a comprehensive and holistic indicator of individuals’ quality of life, influenced by various factors throughout their life course [40]. The present study aimed to examine the role of the theory of gerotranscendence alongside physical, psychological, and social determinants in predicting life satisfaction. The results demonstrated positive associations between physical health, mental health, social health, environmental conditions, and gerotranscendence with life satisfaction.

The results of the present study showed that life satisfaction was positively associated with mental health, such that better mental health among the elderly corresponded to higher life satisfaction. Similarly, Lara et al. (2020) found a relationship between life satisfaction, mental health, and happiness in older adults [41]. This suggests that when an elderly individual perceives their life as meaningful, they tend to experience greater mental well-being and happiness. Additionally, Celik et al. (2018) reported a positive association between life satisfaction and mental health, defining mental health as overall psychological well-being [42]. They identified key symptoms of mental disorders as including feelings of worthlessness, extreme fatigue, loneliness, uselessness, loss of control and significance, depression, anger, and anxiety [43].

Factors such as low levels of education and poverty are also associated with feelings of shame and social withdrawal, all of which can negatively affect mental health and, consequently, life satisfaction [42]. It is likely that individuals with greater life satisfaction have a more positive perspective on life events and are better able to manage the challenges and limitations of aging, which may contribute to improved mental health.

As the current study results showed, the higher the social support an elderly person receives, the greater their sense of pleasure and satisfaction with life. Borhani Nejad and colleagues reported a significant relationship between social participation and life satisfaction in older adults [44]. Similarly, Gilmour’s research indicated that low social participation could lead to life dissatisfaction among the elderly [45]. This may be attributed to participation in collective and family-based activities, as well as the support provided by family members for the elderly’s engagement in the community. Having strong family relationships and receiving the highest level of social support from family can create the foundation for high life satisfaction among older adults [46]. Additionally, Gholizadeh (2010) believes that life satisfaction arises when older adults actively participate socially and their social needs are optimally met [47]. However, the results of the study by Momeni et al. (2018), conducted on 126 elderly participants, showed no significant relationship between social support and life satisfaction [48]. This finding may be attributed to the small sample size and the use of convenience sampling in their study.

A key factor contributing to mental well-being and life satisfaction in older adults is having someone they can rely on for support during times of need [49]. This form of social support enhances an individual’s ability to cope with stress. In fact, older adults who live with their families, maintain social connections, and receive support from family members generally report higher levels of life satisfaction [10]. Family members are consistently the primary source of both instrumental and emotional support, playing a crucial role in promoting the mental health of older adults. A more supportive family environment is strongly and significantly associated with higher life satisfaction. This suggests that the greater the respect and standing an elderly person holds within their family, the greater their overall life satisfaction [50].

A positive relationship between physical health and life satisfaction has been found in the present study. The higher the scores of daily living activities and health among the elderly participants, the greater their life satisfaction. Conversely, greater severity of illness was associated with lower life satisfaction. Pallavi et al. (2015) reported that physical problems such as vision and hearing impairments, difficulties in walking and chewing, speech disorders, and memory issues are major factors affecting life satisfaction [51]. Similarly, Lee et al. (2021) identified physical and mental health as key factors influencing life satisfaction [52]. Good physical health not only facilitates greater participation in activities but also enhances overall life satisfaction. Additionally, it helps foster a positive personal outlook on life [53].

The study demonstrated a relationship between life satisfaction and environmental conditions. These results align with those of Hyun et al. (2016), who reported that elderly individuals living in urban areas and in their own apartments expressed higher life satisfaction [52]. A literature review also indicated that life satisfaction among older adults is linked to physical environmental factors, including adequacy of living facilities, satisfaction with home space and neighborhood, proximity to healthcare services, house size, number of rooms, and the floor of residence [54].

A suitable living environment fosters a sense of security in individuals. According to Maslow’s hierarchy of needs, human needs are divided into five categories: physiological needs, safety, social relationships and affection, esteem, and self-actualization [55]. According to this theory, the need for security is a key social factor influencing life satisfaction and directly affects its level. Furthermore, a strong correlation exists between the sense of security and life satisfaction, both of which are considered important indicators of social health and well-being [52]. Another possible explanation for the link between life satisfaction and the physical environmental conditions is that a suitable environment promotes social interactions and expands the elderly’s social networks, thereby reducing loneliness and enhancing life satisfaction [56].

The results of the study revealed that, beyond other influencing variables, gerotranscendence alone can serve as an independent predictor of life satisfaction among older adults. Since gerotranscendence is a central element of the broader concept of successful aging, these findings are consistent with Zanjari et al., who defined successful aging as encompassing psychological and social health, spirituality and transcendence, physical health, financial security, and an age-friendly social environment [57]. Similarly, Hosseiny et al. (2023) reported a significant relationship between life satisfaction and gerotranscendence in older adults with cancer, supporting the findings of the present study [58].

Yaden et al. (2022) also found that spiritual well-being can significantly predict life satisfaction [59]. Furthermore, transcendence plays a key role in both life satisfaction and psychological adjustment [60]. A systematic review of studies among Muslim populations revealed a consistent positive correlation between religiosity and happiness, highlighting the influence of cultural context on the spirituality–happiness relationship [61].

Kress et al. (2015) found that lower levels of spirituality, religious engagement, life satisfaction, and a sense of meaning in life significantly predict self-harming behaviors and poorer mental health [62]. Similarly, a systematic review found that higher levels of religious involvement are associated with lower rates of depression and suicidal behavior, underscoring the protective role of spirituality [63], findings that are consistent with the present study.

These findings may be explained by the concept of spiritual transcendence, which reflects an individual’s capacity to adopt a broad, reflective perspective on life and derive a deep sense of meaning and purpose. As an intrinsic and fundamental source of motivation, spiritual transcendence encourages individuals to look beyond personal needs and focus on the well-being of others [64]. Consequently, individuals who embody this positive trait often experience greater happiness, higher levels of life satisfaction, and fewer mood-related difficulties. This trait encourages individuals to evaluate their lives through their own unique and personal standards, ultimately fostering a deeper sense of fulfillment and overall life satisfaction [65].

Our findings suggest gerotranscendence is shaped by a complex interplay of factors. Demographically, age and education level appeared as significant correlates, possibly reflecting cumulative life experiences and cognitive resources. Gerotranscendence emphasizes a different and positive view of aging in which older adults can be understood with consideration as a whole person, a position which is in line with the concept of active aging. However, Tornstam and other scholars have supported this view through qualitative and quantitative studies [66,67,68]. Also this concept is related to cultural, psychological, social, and religious contexts. Psychologically, mental well-being and cognitive function provided the foundation for transcendent perspectives. Socially, robust support networks facilitated the re-evaluation of relationships central to gerotranscendence. Spiritually and culturally, the Iranian context with its rich philosophical and religious traditions may uniquely shape transcendent development. In conclusion, incorporating gerotranscendence into aging policies and practices has the potential to profoundly enhance the quality of life of older adults, ultimately creating a more compassionate and meaningful experience of aging [69].

Although evidence supports a positive association between spirituality, life satisfaction, and mental health, it is important to recognize that these benefits may not extend to everyone. For some individuals, conflicts between spiritual beliefs and mental health challenges may arise, highlighting the need for personalized and culturally sensitive approaches in mental health care.

While our model demonstrates strong predictive power in community-dwelling older adults, its application to specific populations warrants careful consideration. For older adults with significant comorbidities, the physical health components may exert a more dominant influence on life satisfaction, potentially altering the relative weights of gerotranscendence and other psychosocial factors. Similarly, for institutionalized older adults, environmental factors and social support networks may play fundamentally different roles compared to community settings. Future research should validate this model in these specific subpopulations and explore potential modifications to account for their unique circumstances.

This study had several important limitations. First, its exclusive focus on older adults living in Tehran restricts the generalizability of the findings to other cities, necessitating cautious interpretation in light of the socio-cultural and economic diversity across regions. Second, the exclusion of elderly individuals with severe illnesses (e.g., cancer) may have resulted in an incomplete representation of the population’s overall health status. Third, the reliance solely on self-reported medical conditions, without corroboration from medical records, introduces the risk of recall bias, potentially leading to under or overestimation of health issues. Fourth, the cross-sectional design limits the ability to draw causal conclusions. Finally, although this study sought to achieve satisfactory population representation through the use of a systematic sampling frame (telephone lists) and random selection of participants, the sampling method should be considered a limitation. The use of telephone lists may have limited access to certain subgroups of older adults (such as those without landline telephones or those who declined phone communication), which could somewhat affect the generalizability of the results. These limitations highlight the need for future longitudinal research with more diverse samples and mixed-methods designs, such as integrating clinical data with self-reported measures, to yield a more comprehensive understanding.

## 5. Conclusions

The study found that elderly participants reported above-average levels of life satisfaction and gerotranscendence. Although the proposed model demonstrates strong predictive power for community-dwelling older adults, its applicability to those with significant comorbidities or in institutional settings requires further investigation. Future research should explore the nuanced interactions between demographic, psychological, social, spiritual, cultural, and biographical factors in shaping gerotranscendence across diverse populations. Healthcare providers and policymakers should consider these multidimensional influences when designing interventions to enhance late-life development and well-being. Furthermore, our analysis revealed that the expression of gerotranscendence is likely deeply interwoven with the specific cultural, spiritual, and familial fabric of the Iranian context, suggesting that while the core theory is universal, its manifestation is culturally nuanced. Future research should employ longitudinal and mixed-methods designs to further explore the causal pathways uncovered in this model and to validate these intervention strategies across diverse cultural and clinical populations.

## Figures and Tables

**Table 1:** Association Between Life Satisfaction and Physical, Mental, Social, and Environmental Health Variables Among the Elderly Participants.

	Instruments Used	Mean	Standard Deviation	Lowest Value	Highest Value	Lowest Value	Highest Value	R	*p*-Value
Physical health	Self-reported overall health (SRH)	2.17	0.84	1	5	1	5	−1.170	*** 0.001
	Charlson Comorbidity Index (CCI)	1.8	1.9	0	37	0	13	0.19	*** 0.001
	Activity of Daily Living (ADL)	10.7	3.17	0	12	0	12	0.36	*** 0.001
Mental health	World Health Organization 5-item Well-being Index (WHO-5)	11.6	5.87	0	25	0	25	0/550	*** 0.001
	Abbreviated Cognitive Test (AMT)	8.29	2.29	0	10	0	10	0.22	*** 0.001
Social health	Sherbourne and Stewart Social Support (MOS-SSS)	70	15.3	19	95	19	95	0.26	*** 0.001
	Lubben Social Network (LSN-6)	13.34	5.74	0	30	0	30	0.25	*** 0.001
Environmental condition	Environmental health status according to the World Health Organization (WHOQOL)	12.5	2.4	4	20	4	20	0.4	*** 0.001
Gerotranscendence	Gerotranscendence Scale	74.11	6.34	25	100	45	96	0.35	*** 0.001
Life satisfaction	Form Z Life Satisfaction Questionnaire (LS13-Z)	12.78	4.86	0	26	0	25		

*** *p* < 0.001. Note. SRH = Self-Rated Health; CCI = Charlson Comorbidity Index; ADL = Activities of Daily Living; WHO-5 = World Health Organization Five Well-Being Index; AMT = Abbreviated Mental Test; MOS-SSS = Medical Outcomes Study Social Support Survey; LSN-6 = Lubben Social Network Scale; WHOQOL = World Health Organization Quality of Life.

**Table 2:** Results of Hierarchical Regression Analysis.

		The First Block			The Second Block			The Third Block		
		**β**	**T**	***p*-Value**	**β**	**T**	***p*-Value**	**β**	**T**	***p*-Value**
Demographic information	Age	0.005	0.12	0.9	0.03	0.77	0.44	0.04	1.18	0.24
	Gender	−0.030	−0.680	0.49	−0.004	−0.090	0.93	0.001	0.008	0.99
	Ethnicity	0.04	0.09	0.36	−0.010	−0.320	0.74	−0.004	−0.10	0.92
	Marital status	0.06	35.1	0/180	0.06	1.7	0.09	0.06	1.76	0.08
	Literacy	0.08	1.89	0.05	−0.008	−0.220	0.82	−0.02	0.51	0.61
	Status of residence	0.02	0.57	0.56	−0.008	−0.240	0.08	−0.001	−0.020	0.98
	Employment Status	0.08	2	0.05	0.04	1.02	0.31	0.03	1	0.32
	The state of income	0.05	1.1	0.27	−0.060	−1.730	0.8	−0.06	−1.850	0.06
Physical health	Charlson Comorbidity Index (CCI)				−0.020	−0.620	0.53	−0.02	−0.550	0.58
	Activity of Daily Living (ADL)				−0.040	−1.170	0.24	−0.020	0.55	0.58
	Self-reported overall health (SRH)				0.06	1.42	0.16	0.06	1.54	0.12
Mental health	World Health Organization 5-item Well-being Index (WHO-5)				0.41	10.11	0.001	0.36	8.79	0.001
	Abbreviated Cognitive Test (AMT)				0.05	1.3	0.19	0.04	1.11	0.26
Social health	Sherbourne and Stewart Social Support (MOS-SSS)				0.04	1.19	0.23	0.04	1.15	0.25
	Lubben Social Network (LSN-6)				0.09	2.42	0.02	0.08	2.4	0.02
Environmental condition	Environmental health status according to the World Health Organization (WHOQOL)				0.19	4.84	0.001	0.17	4.38	0.001
(Gerotranscendence)								0.17	4.94	0.001
F		3.13			21.44			22.43		
*p*-value		0.002			0.001			0.001		
R Square		0.04			0.37			0.04		
F Change		3.13			38.16			24.47		
R square change		0.04			0.33			0.02		

Note. SRH = Self-Rated Health; CCI = Charlson Comorbidity Index; ADL = Activities of Daily Living; WHO-5 = World Health Organization Five Well-Being Index; AMT = Abbreviated Mental Test; MOS-SSS = Medical Outcomes Study Social Support Survey; LSN-6 = Lubben Social Network Scale; WHOQOL = World Health Organization Quality of Life.

**Table 3:** Selected Indices for Evaluating the Goodness-of-Fit of the Proposed Mode.

Index	Calculated Value for the Hypothetical Model	Acceptable Value for Confirming Model Fit	Result
**CMIN**	2.545	The value of 1 and 3 (less than 5)	A good confirmation
**RSMEA**	0.051	Smaller than 0.08	A good confirmation
**GFI**	0.815	More than 0.9	Not good confirmation
**PCLOSE**	0.272	More than 0.05	A good confirmation

Note: CMIN Minimum Discrepancy Function, RSMEA Root Mean Square Error of Approximation, GFI Goodness-of-Fit Index, PCLOSE *p*-Value of Close Fit.

## Data Availability

Data supporting the findings of this study are available upon reasonable request to the corresponding author due to ethical and legal constraints.

## Abbreviations

The following abbreviations are used in this manuscript:

ML	Maximum Likelihood
SEM	Structural Equation Modeling
LSI-Z	Life Satisfaction Index-Z
CCI	Charlson Comorbidity Index
ADL	Activities of Daily Living
WHO-5	World Health Organization Five Well-Being Index
AMT	Abbreviated Mental Test
MOS-SSS	Medical Outcomes Study Social Support Survey
LSN-6	Lubben Social Network Scale
WHOQOL-BREF	World Health Organization Quality of Life—BREF
